# Electroacupuncture mediates extracellular signal-regulated kinase 1/2 pathways in the spinal cord of rats with inflammatory pain

**DOI:** 10.1186/1472-6882-14-285

**Published:** 2014-08-04

**Authors:** Jian-Qiao Fang, Jun-Fan Fang, Yi Liang, Jun-Ying Du

**Affiliations:** Department of Neurobiology & Acupuncture Research, the Third Clinical College, Zhejiang Chinese Medical University, 548 Binwen Road, Binjiang District, Hangzhou, Zhejiang Province 310053 China

**Keywords:** Electroacupuncture, Analgesia, Inflammatory pain, CFA, ERK1/2, CREB, NK-1, COX-2

## Abstract

**Background:**

Activation of extracellular signal-regulated kinase1/2 (ERK1/2) in dorsal horn of the spinal cord by peripheral inflammation is contributed to inflammatory pain hypersensitivity. Although electroacupuncture (EA) has been widely used to alleviate various kinds of pain, the underlying mechanism of EA analgesia requires further investigation. This study investigated the relationship between EA-induced analgesia and ERK signaling involved in pain hypersensitivity.

**Methods:**

The rats were randomly divided into control, model, EA and sham EA groups. Inflammatory pain model was induced by injecting of 100 μl Complete Freund’s adjuvant (CFA) into the plantar surface of a hind paw. Rats in the EA group were treatment with EA (constant aquare wave, 2 Hz and 100 Hz alternating frequencies, intensities ranging from 1-2 mA) at 5.5 h, 24.5 h and 48.5 h. Paw withdrawal thresholds (PWTs) were measured before modeling and at 5 h, 6 h, 25 h and 49 h after CFA injection. Rats were killed and ipsilateral side of the lumbar spinal cords were harvested for detecting the expressions of p-ERK1/2, Elk1, COX-2, NK-1 and CREB by immunohistochemistry, real-time PCR, western blot analysis and EMSA. Finally, the analgesic effect of EA plus U0126, a MEK (ERK kinase) inhibitor, on CFA rats was examined.

**Results:**

Inflammatory pain was induced in rats by hindpaw injection of CFA and significantly increased phospho-ERK1/2 positive cells and protein levels of p-ERK1/2 in the ipsilateral spinal cord dorsal horn (SCDH). CFA up-regulated of cyclooxygenase-2 (COX-2) mRNA and protein expression at 6 h after injection and neurokinin-1 receptor (NK-1) expression at 49 h post-injection, in the SCDH. EA, applied to Zusanli (ST36) and Kunlun (BL60), remarkably increased the pain thresholds of CFA injected rats, significantly suppressed ERK1/2 activation and COX-2 protein expression after a single treatment, and decreased NK-1 mRNA and protein expression at 49 h. EA decreased the DNA binding activity of cAMP response element binding protein (CREB), a downstream transcription factor of ERK1/2, at 49 h after CFA injection. Moreover, EA and U0126 synergistically inhibited CFA-induced allodynia.

**Conclusions:**

The present study suggests that EA produces analgesic effect by preventing the activation of ERK1/2-COX-2 pathway and ERK1/2-CREB-NK-1 pathway in CFA rats.

## Background

Currently, inflammatory pain is one of the most serious health problems worldwide. It is generally treated with opioids and non-steroid anti-inflammatory drug (NSAID), but both are limited by side effects [1]. Acupuncture has long been used for the treatment of various diseases in China, with few side effects. Electroacupuncture (EA), an alternative way of administering acupuncture, refers to the application of a pulsating electrical current to acupuncture needles for acupoint stimulation, and is a potential treatment for inflammatory pain [2, 3]. Although many studies, including our own [4, 5], showed that EA produced anti-allodynia in patients and animal models of inflammatory pain [6], the mechanisms of EA analgesia are still unclear.

Extracellular signal-regulated kinase1/2 (ERK1/2) is a member of the mitogen-activated protein kinases (MAPKs) family. Although the exact mechanism of inflammatory pain is still unclear, there is mounting evidence demonstrating MAPKs family play an important role in its pathphysiology [7–10], especially ERK1/2 activated in superficial dorsal horn [11]. It has been well demonstrated that ERK1/2 regulates expressions of neurokinin-1 receptors (NK-1) and cyclooxygenase-2 (COX-2) [12, 13], two important mediators of inflammatory pain in central nervous system [14, 15], and thus contributed to altering sensitivity to pain. In order to regulate gene expression, ERK1/2, as a signaling molecule, must be translocated to the nucleus to phosphorylate nuclear substrates such as Ets-like kinase 1 (Elk1) and cAMP response element binding protein (CREB) [16, 17]. These findings suggest that ERK1/2 signal pathway (ERK1/2-Elk1/CREB-COX-2/NK-1) in the dorsal horn may be a target for inflammatory pain treatment.

In previous study, we found that analgesic effect of transcutaneous electrical nerve stimulation (TENS) on inflammatory pain partly produced by modulating the activation of the spinal ERK1/2 [18]. Our study also indicated that EA’s analgesic effect was associated with the inhibition of the activation of ERK1/2 in the spinal cord dorsal horn (SCDH) in a rat inflammatory pain model [19]. Furthermore, ERK1/2 signal pathway has been regulated by EA stimulation in central nerve system [20]. It has been demonstrated that intracellular signal transduction pathways in spinal cord, such as p38 MAPK pathway [21], have a crucial role in EA analgesia, however, few reports detected that whether EA alleviated inflammatory pain by modulating ERK signal transduction pathway activity in the superficial spinal cord. Thus, we used a rat inflammatory pain model to investigate the hypothesis that ERK signal transduction pathway is involved in EA analgesia.

## Methods

### Animals

All animal care, surgery, and handling procedures approved by the animal experiment centre attached to Zhejiang Chinese Medical University were performed strictly in accordance with the National Institutions of Health Guide for the Care and Use of Laboratory Animals. Adult Male Sprague–Dawley rats (200–230 g), used for this study, were obtained from the animal experiment centre attached to Zhejiang Chinese Medical University. They were housed with artificial 12-h light–dark cycle lighting with a controlled temperature (23 ± 1°C), and relative humidity (70 ± 10%), and distilled water and food were available *ad libitum*.

### Experimental design

Two sets of experiments were conducted to investigate the involvement of the ERK signal pathway in EA anti-allodynia: (I) EA regulated ERK signal pathway (II) EA plus ERK antagonists. In experiment I, 200 rats were randomly divided into four groups: (a) a control group receiving normal saline (NS) injection, adminstered by the same route and with the same volume as Complete Freud’s Adjuvant (CFA, Sigma, USA) injection (n = 20); (b) a model group receiving 100 μl CFA injection into the plantar surface of the right hind paw (n = 60); (c) an EA group receiving CFA injection and EA treatment (n = 60) and (d) a sham EA group receiving CFA injection and sham EA treatment (n = 60). At 6 h, 25 h and 49 h after modeling, 20 rats of each group for model, EA and sham EA were sacrificed to detecte the expression of ERK1/2 and its downstream molecules by immunohistochemistry (n = 6), western blot (n = 6), real-time PCR (n = 6) and electrophoretic mobility shift assay (EMSA) (n = 2). Rats in control group were only sacrificed at 49 h after CFA injection. In experiment II, 30 rats were injected with CFA and randomly divided into three groups: (a) EA plus 10% dimethyl sulfoxide (DMSO) (n = 10); (b) EA plus MEK (ERK kinase) inhibitor (U0126) in 10% DMSO (n = 10); (c) 10% DMSO alone (n = 10). In both experiments, 10 rats from each group were randomly chosen for behavioral testing. After CFA injection, rats were returned to their cage and allowed to recover.

### Drug administration

For intrathecal drug delivery, a polyethylene-10 catheter was implanted into the intrathecal space of the spinal cord at the lumbar enlargement. The animals were allowed to recover for 7 days after the operation before experimentation. Rats were loosely immobilized by an assistant’ s hands and 10 μl U0126 (1 μg; dissolved in 10% DMSO), as previously described [12], was administrated at 5.5, 24.5 and 48.5 h after CFA injection. DMSO (10%, Abcam, UK) was injected as a vehicle control.

### Behavioral analysis

All pain behavioral testing was performed by a trained investigator who was blinded to the experimental conditions. Inflammatory allodynia was defined as a decrease in paw withdrawal thresholds (PWTs) to a non-noxious mechanical stimulus. PWTs were tested with an automated von Frey-type testing device (Dynamic Plantar Aesthesiometer 37450, UGO Basile, Italy) as described previously [21]. Rats received two training sessions before the experiment started. Rats were placed on a metal mesh table and adapted to the new environment for 30 min. The mechanical stimulus was delivered to the plantar surface of the right hind paw below the floor of the plastic test chamber. A steel rod (diameter of 0.5 mm) was pushed against the hind paw with ascending force. The force went from 0 to 50 g over a 20 s period. When the animal withdrew its hind paw, the mechanical stimulus was automatically stopped and the force at which the rat withdrew its paw was recorded to the nearest 0.1 g. In both experiments, the mechanical touch sensitivity of paws were measured before modeling, and 5, 6, 25 and 49 h after CFA administration.

### EA treatment

The EA or sham EA stimulation was applied at 5.5, 24.5 and 48.5 h after CFA injection. Rats were loosely immobilized by an assistant’ s hands during the whole treatment. Four stainless steel acupuncture needles of 0.25 mm in diameter were inserted at a depth of 5 mm into the bilateral “Zusanli” (ST36, between the tibia and fibula 5 mm below the knee) and “Kunlun” (BL60, 3 mm proximal to the lateral malleolus) acupoints. The two ipsilateral needles were connected to the output terminals of the HANS Acupuncture point Nerve Stimulator (LH-202H, Huawei Co., Ltd., Beijing, China). Electro-stimulation was delivered with constant parameters, constant square wave current output (pulse width: 0.6 ms at 2 Hz, 0.2 ms at 100 Hz); intensities ranging from 1–2 mA (each intensity for 15 min, totaling 30 min); at 2 Hz and 100 Hz alternating frequencies (automatically shifting between 2 Hz and 100 Hz stimulation for 3 s each). Sham EA group animals received the same subcutaneous needle insertion (2 mm in depth) into ST36 and BL60, and the needles were linked to the electrodes but without electrical stimulation. To eliminate the stress effect, rats in the model and sham EA groups were also loosely immobilized by an assistant’ s hands similar to the EA group.

### Immunohistochemistry

Rats were deeply anesthetized with 10% choral hydrate (0.35 ml/100 g, intraperitoneal [i.p.]) and perfused through the ascending aorta with saline, followed by 4% paraformaldehyde. Lumbar spinal cord (L4-L6) segments were dissected and post-fixed for 6 h with the same fixative solution. Transverse spinal cord sections (free floating, 30 μm) were cut and processed for immunohistochemistry using the streptavidin-perosidase (SP) method. Briefly, endogenous peroxidase activity was quenched with a 15 min incubation in 3% H_2_O_2_ at 37°C. Sections were blocked with 5% goat serum plus 0.1% Triton X-100 for 1 h at 37°C and incubated overnight at 4°C with primary antibody. The sections were then incubated for 1 h with biotinylated secondary antibody (1:200) and 1 h with horseradish peroxidase (HRP)-conjugated-avidin at 37°C. Finally, the reaction product was visualized with diaminobenzidine (DAB) in 0.1 M phosphate-buffered saline (PBS) for 30 s. Sections were mounted on glass slides, dehydrated through an ascending series of alcohol, cleared with xylene, and coverslipped. Images were captured from the ipsilateral dorsal horn at 20× magnification using a Leica CCD camera. The following antibodies were used: anti-phospho-ERK1/2 (p-ERK1/2) (1:400, CST, USA) and anti-phospho-Elk1 (p-Elk1) (1:400, CST, USA). Five nonadjacent sections from the L4-L6 lumbar spinal cord were randomly selected, and the numbers of immunoreactive (IR) cells in the superficial lamina (lamina I-II) of the dorsal horn in each section were counted by an observer blind to the treatment. The mean values from the five sections were determined for each animal.

### Western blot

All rats were anesthetized with 10% choral hydrate (0.35 ml/100 g, i.p.) and perfused transcardially with 150 ml cold sterilized saline. Ipsilateral spinal dorsal horns (L4-L6) were removed and preserved at -80°C. For western blot, the dorsal horns were homogenized with a mechanical rotary cutter in strong RIPA buffer containing a cocktail of phosphatase inhibitors and proteinase inhibitors. The extracted protein was boiled in a sodium dodecyl sulfate (SDS) sample buffer (100 mm Tris, pH 6.8, 2% SDS, 20% glycerol, 10% β-mercaptoethanol, and 0.1% bromophenol blue). Protein samples were separated by SDS-PAGE gel and transferred to nitrocellulose membranes. The filters were blocked with 5% low-fat milk and incubated overnight at 4°C with primary antibody. Then the bolts were incubated for 1 h at 37°C with HRP-conjugated secondary antibody (1:10,000) and visualized in ECL solution (Thermo Scientific, USA) for 1 min and exposed by the ImageQuant Las 4000 (General Electric company). The band densities were quantified with a computer-assisted imaging analysis system (Image Quant software) and β-actin was used as an internal control. The following primary antibodies were used: anti-phospho-ERK1/2 (p-ERK1/2) (1:2000, CST, USA), anti-COX-2 (1:1000, Caymen Chemical company, USA), anti-NK-1 (1:1000, Millipore, USA), and anti-β-actin (1:1000, CST, USA).

### Real-time PCR

All rats were anesthetized with 10% choral hydrate (0.35 ml/100 g, i.p.) and perfused transcardially with 150 ml cold sterilized saline. Ipsilateral spinal dorsal horns (L4-L6) were removed and preserved at -80°C. Total RNA was extracted using Trizol Reagent (Invitrogen, France) by the guanidium thiocyanate method according to the manufacturer’s instructions. RNA was quantified by spectrophotometry. First strand cDNA was synthesized from 1 μg of total RNA in a final volume of 10 μl using the PrimerScript® RT reagent Kit with gDNA Eraser (Takara, Japan). Relative mRNA levels were quantified by RT-PCR using the fluorescent EvaGreen technology. cDNA (1.5 μl) was subjected to real-time quantitative PCR using the CFX96™ real-time PCR detection system (Bio-Rad, USA). Primer premier 5.0 software (Premier, Canada) was employed to design oligonucleotide primers specific for rat COX-2, NK-1 and GAPDH (an internal control). COX-2, forward: 5′-CACGGACTTGCTCACTTTGTT-3′, reverse, 5′-AAGC GTTTGCGGTACTCATT-3′; NK1, forward, 5′-CCACCAACACCTCTGAGTCTAA CC-3′, reverse, 5′-GCCAGGTCCACCAGGAAATAA-3′; GAPDH, forward, 5′-TGCTGAGTATGTCGTGG AG-3′, reverse, 5′-GTCTTCTGA GTGGCAGTGAT-3′, with product sizes of 161basic pairs (bp), 178 bp, and 288 bp, respectively. Reactions (total volume, 20 μl) were incubated at 95°C for 30 min, followed by 40 cycles of 10 s at 95°C and 30 s at 55.9°C (COX-2) or 59.0°C (NK-1). Water controls were included to ensure specificity. Each sam-ple was measured in triplicate, and data points were examined for integrity by analysis of the amplification plot. Adding the melting curve analysis in the reaction condition, the analytical model: 65°C-95°C, an increase of 0.5°C every 10 s. The comparative cycle threshold Ct method was used for the relative quantification of gene expression. The amount of COX-2 and NK-1 mRNA, normalized to GAPDH and relative to a calibrator, was given by 2^-ΔΔCt^, with Ct indicating the cycle number at which the fluorescence signal of the PCR product crosses an arbitrary threshold set within the exponential phase of the PCR, and ΔΔCt = [(Ct_target (unknown sample)_-Ct_end.control (unknown sample)_)]-[(Ct_target (calibrator sample)_-Ct_end.control (calibra®or sample)_)].

### Electrophoretic mobility shift assay

All rats were anesthetized with 10% choral hydrate (0.35 ml/100 g, i.p.) and perfused transcardially with 150 ml cold sterilized saline. Ipsilateral spinal dorsal horns (L4-L6) were removed and preserved at -80°C. Nuclear protein was extracted from spinal cord dorsal horn according to the manufacturer’s instructions of a Nuclear Extract kit (Thermo Fisher Scientific, USA). For the CREB promoter, the two complementary consensus oligonucleotides were: 5′-AGAGATTGCCTGACGTCAGAGAGCTAG-3′, 5′-CTAGCTCTTGACGTCAGGCAATCTCT-3′, and the two complementary mutant oligonucleotides were: 5′-AGAGATTGCCTG*TG*GTCAGAGAGCTAG-3′, 5′-CTAGCTCT*CT*GACCACAGGCAATCTCT-3′. Oligonucleotides 3′- were labeled by biotin. To generate double-stranded probes, biotin-labeled complementary oligonucleotides were boiled for 5 min in 1 × sodium-Tirs-EDTA buffer (STE, 10 mM Tris–HCl and 1 mM EDTA, pH 8.0), then cooled slowly to room temperature overnight. Unlabeled complementary oligonucleotide pairs were annealed to make double-stranded competitor probes. EMSA was performed using a Light Shift Cheniluminescent EMSA kit (Thermo Fisher Scientific, USA). Ten μg of unclear protein from each sample was incubated with a biotinylated probe in the reaction buffer for 30 min at room temperature (RT). Then the reaction mixture was subjected to 6% non-denaturing polyacrylamide gel electrophoresis, and transferred to Nylon membranes. Biotin-labeled DNA was detected using a streptavidin-horseradish peroxidase conjugate and chemiluminescent ECL kit (Thermo Fisher Scientific, USA). The images were captured by ImageQuant Las 4000 and analyzed using ImageQuant software. A competition EMSA was performed by adding 100× molar excess of unlabeled doubled-stranded oligonucleotide.

### Quantification and statistics

Differences between groups were compared using student’s *t*-test or ANOVA, followed by least significant difference (LSD) test. The data were represented as mean ± standard error mean (SEM). The PWTs data were normally distributed, and were therefore analyzed using a repeated-measures analysis of variance (ANOVA) with between-subjects factors. The density of specific bands of western blotting was quantified by densitometric scanning analysis. The criterion for statistical significance was *P* < 0.05.

## Results

### Effect of EA on inflammatory pain hypersensitivity in CFA rats

**Figure 1:**
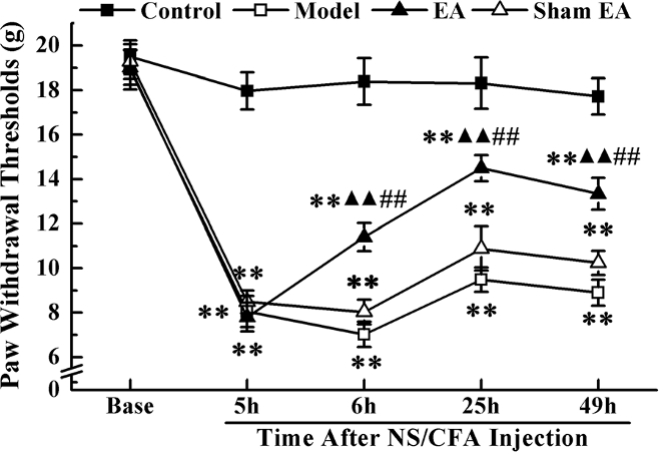
**The paw withdrawal thresholds (PWTs) of rats in each group at different time-points.** PWTs were measured at pre-injection, 5, 6, 25 and 49 h after NS/CFA injections. Values represent mean ± SEM; n = 10 per group for each time point. **P < 0.01 versus control group at the corresponding time point. ^▲▲^ *P* < 0.01 versus model group at the corresponding time point. **##** *P* < 0.01 versus sham EA group at the corresponding time point.

Mean PWTs in all experimental groups at each time point are shown in Figure 1. The repeated-measures ANOVA with between-subjects factors revealed differences over time points (*P* < 0.01) and between groups (*P* < 0.01). There were significant interactive effects between time points and groups (*P* < 0.01). *Post-hoc* LSD tests indicated that CFA injection caused decreased PWTs in rats (*P* < 0.01). Sham EA treatment did not change rat PWTs compared with the model group (*P* > 0.05). A significant increase in PWTs was observed in the EA group, when compared with the model group (*P* < 0.01) and the sham EA group (*P* < 0.01). However, PWTs in the EA group still showed a significant difference compared with those of the control group (*P* < 0.01).

To compare PWTs between groups at different time points, model and sham EA groups were combined because there was no significant difference in PWTs between them. One-way ANOVA for independent samples identified that significant differences occurred among controls, EA and combined groups from 6 h to 49 h after CFA injection (*P* < 0.01). Compared with the control group, plantar injection of CFA to the right hind paw significantly decreased the mechanical threshold during the observed time points (*P* < 0.01). Following EA treatment, PWTs of rats in the EA group were significantly increased compared with those in the model and sham EA group from 6 h to 49 h (*P* < 0.01). However, at 49 h after CFA injection, a significant difference in PWTs was still observed between the EA and control groups (*P* < 0.01).

### P-ERK1/2, COX-2 and NK-1 induction by peripheral inflammation

**Figure 2:**
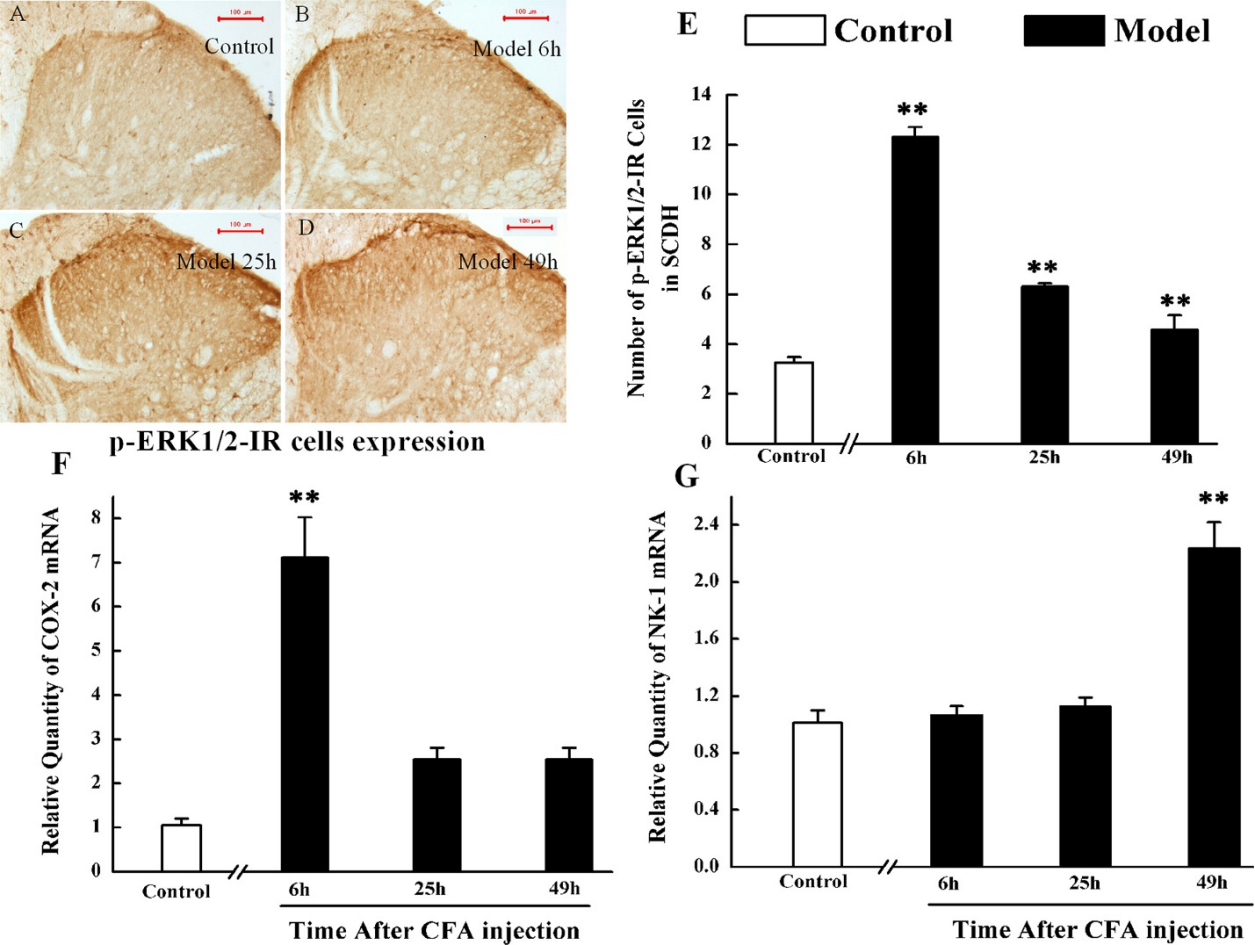
**The expressions of p-ERK1/2-IR cells, COX-2 mRNA and NK-1mRNA in spinal cord dorsal horn of rats in control group and at 6, 25 and 49 h after subcutaneous injection of CFA into the plantar hindpaw region. A-D**. Immunohistochemistry shows p-ERK1/2-IR cells in the L4-6 ipsilateral spinal cord dorsal horn in control rats **(A)**, model rats at 6 h **(B)**, 25 h **(C)**, and 49 h **(D)**. **E-G**. Quantification of p-ERK1/2-IR cells, COX-2 mRNA and NK-1 mRNA expression in L4-6 ipsilateral spinal cord dorsal horn, respectively. Results are mean ± SEM; n = 6. *P < 0.05, **P < 0.01 versus control group at the corresponding time point. The scale bar is 100 μm.

As shown in Figure 2A-D, peripheral inflammation induced by CFA injection resulted in the activation of ERK1/2 in the superficial dorsal horn on the ipsilateral side of the lumbar enlargement. The numbers of phospho-ERK1/2 (p-ERK1/2)-IR cells were increased from 6 h to 49 h after CFA injection (*P* < 0.01) (Figure 2E). We further investigated the expressions of COX-2 and NK-1 mRNA, two commonly detected markers of pain conditions in a variety of pain models [22, 23], at different time points. Real-time PCR was used to measure their expression in response to intraplantar CFA injection. The over-expression of COX-2 mRNA in SCDH was only detected at 6 h after CFA treatment (*P* < 0.01) (Figure 2F), and a mild up-regulation of COX-2 mRNA in SCDH was detected at 25 and 49 h after CFA injection (*P* > 0.05). In contrast to COX-2 mRNA expression, CFA injection significantly increased NK-1 mRNA expression only at 49 h after CFA injection (*P* < 0.01) (Figure 2G).

### Effect of EA on ERK1/2 activation in the lumbar spinal cord

**Figure 3:**
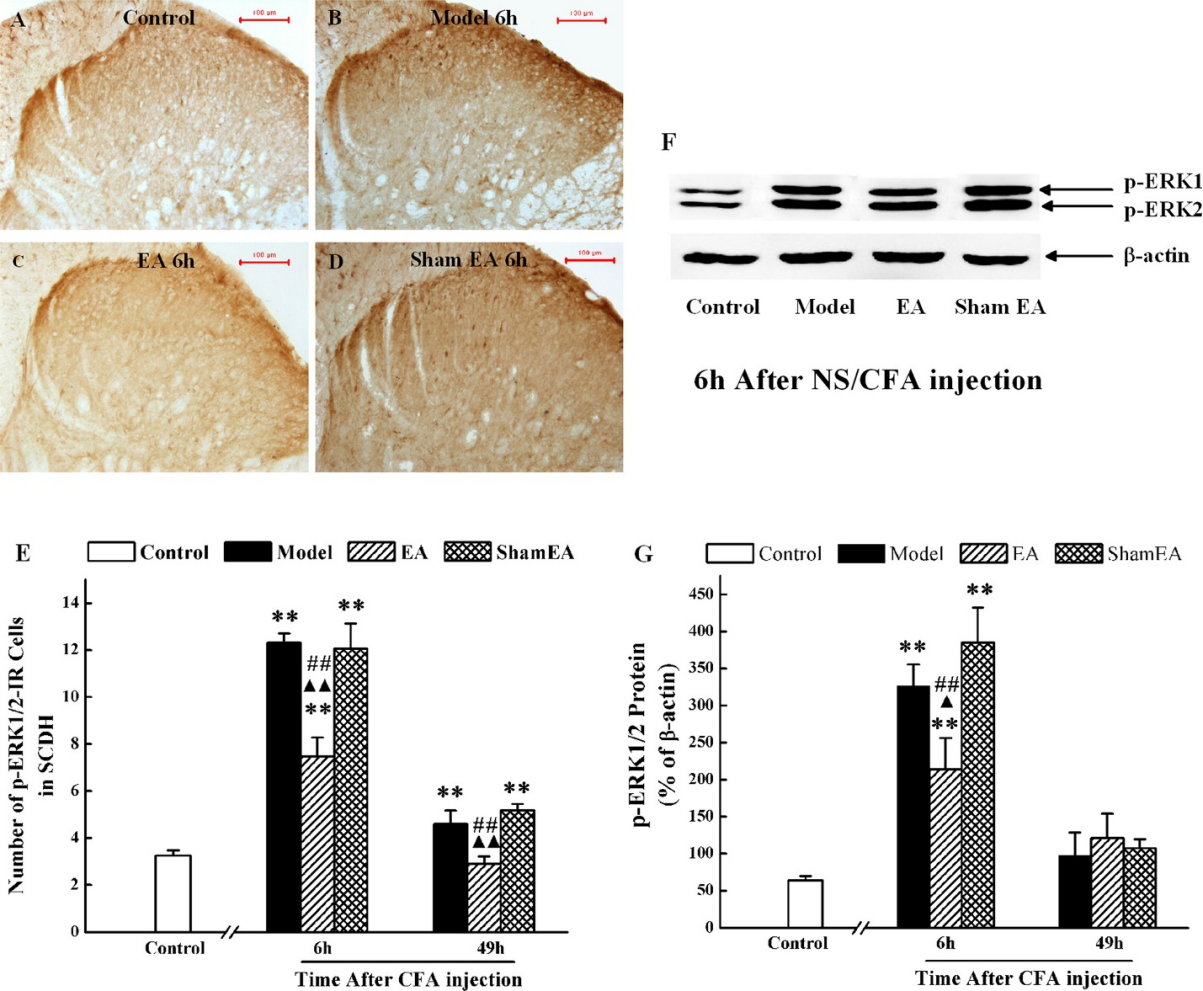
**Effect of EA on p-ERK1/2 expression in the spinal cord dorsal horn in inflammatory pain.** Immunohistochemiscal analysis shows p-ERK1/2-IR cells in the L4-6 ipsilateral spinal cord dorsal horn in control rats **(A)**, model rats **(B)**, EA-treated rats **(C)** and sham EA-treated rats **(D)** at 6 h after CFA injection. **(E)**. Quantification of p-ERK1/2-IR cells in the L4-6 ipsilateral spinal cord dorsal horn of the control group and at 6 and 49 h after CFA injection. **(F)**. The expression of p-ERK1/2 protein in the L4-6 ipsilateral spinal cord dorsal horn by western blot. **(G)**. The quantification of p-ERK1/2 protein normalized against β-actin. Results are mean ± SEM; n = 6. **P < 0.01 versus control group at the corresponding time point. ^▲^ *P* < 0.05, ^▲▲^ *P* < 0.01 versus model group at the corresponding time point. **##** *P* < 0.01 versus sham EA group at the corresponding time point. The scale bar is 100 μm.

Based on the above findings, we tested the effect of EA on ERK1/2 activation at 6 and 49 h after CFA injection using immunohistochemistry. The CFA-evoked increase of numbers of p-ERK1/2-IR cells in the superficial SCDH was suppressed by EA treatment at 6 h and 49 h after CFA injection (*P* < 0.01) (Figure 3A-E). We also investigated the effect of EA on p-ERK1/2 protein expression at 6 and 49 h according to the over-expressing time points of COX-2 and NK-1. Different with the immunohistochemistry results (Figure 2E), p-ERK1/2 protein was not highly expressed at 49 h after CFA injection, compared with the control level (Figure 3G). Compared with the model group, EA only reduced CFA-increased p-ERK1/2 protein levels in the ipsilateral dorsal horn at 6 h after CFA-injection (*P* < 0.01) (Figure 3F-G). Sham EA administration did not affect either the number of labeled cells or p-ERK1/2 protein expression at 6 h and 49 h, when compared with the model group (*P* > 0.05, Figure 3).

### Effect of EA on COX-2 expression

**Figure 4:**
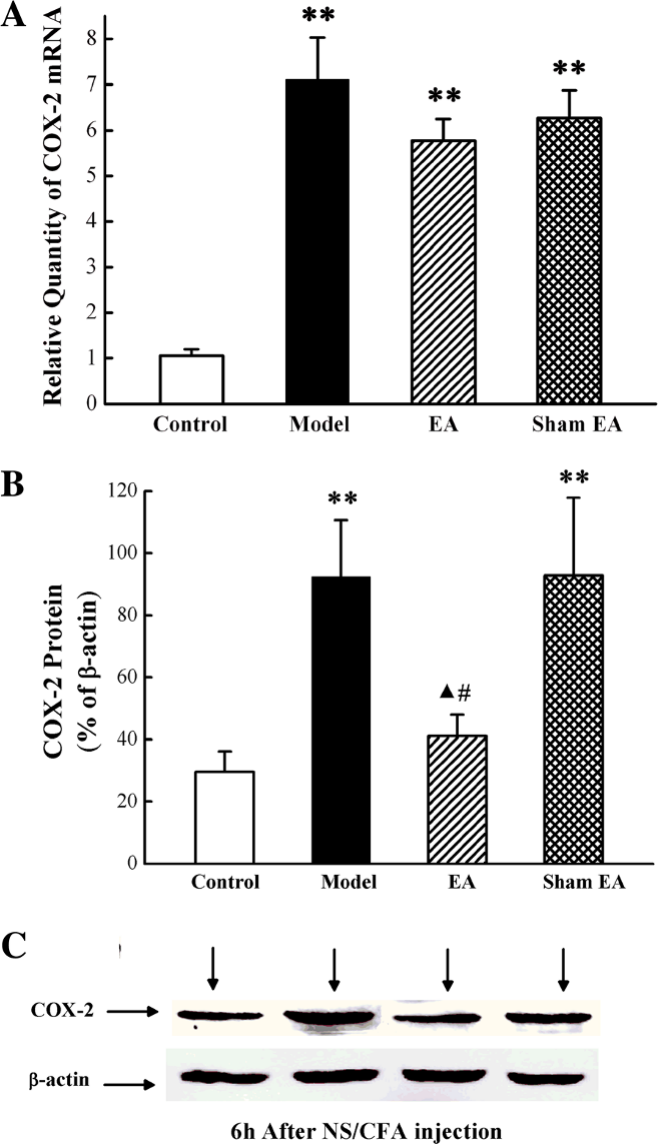
**Effect of EA on COX-2 expression in the ipsilateral spinal dorsal horn at 6 h after CFA injection. (A)**. The expression of COX-2 mRNA (relative to GAPDH) in L4-6 ipsilateral spinal dorsal horn was measured by real-time PCR. **P < 0.01 versus control group. ^▲^ *P* < 0.05 versus model group. **#** *P* < 0.05 versus sham EA group. Results are mean ± SEM; n = 6. **(B)**. The expression of COX-2 protein measured by western blotting analysis. **P < 0.01 versus control group. ^▲^ *P* < 0.05 versus model group. **#** *P* < 0.05 versus sham EA group. Results are mean ± SEM; n = 6. **(C)**. COX-2 protein in normalized against β-actin.

To investigate whether EA could regulate COX-2 expression, we used both real-time PCR and western blot to measure the COX-2 mRNA and protein at 6 h after CFA treatment. Both COX-2 mRNA and protein expression in SCDH significantly increased at 6 h after CFA injection, compared with the control group (*P* < 0.01). No significant difference in COX-2 mRNA expression was observed at 6 h after CFA injection (*P* > 0.05) between the model, EA and sham EA groups (Figure 4A). However, EA treatment significantly inhibited CFA-induced over-expression of COX-2 protein (*P* < 0.01), while sham EA treatment did not show any inhibitory effect on COX-2 protein (*P* < 0.01) (Figure 4B).

### Effect of EA on NK-1 expression

**Figure 5:**
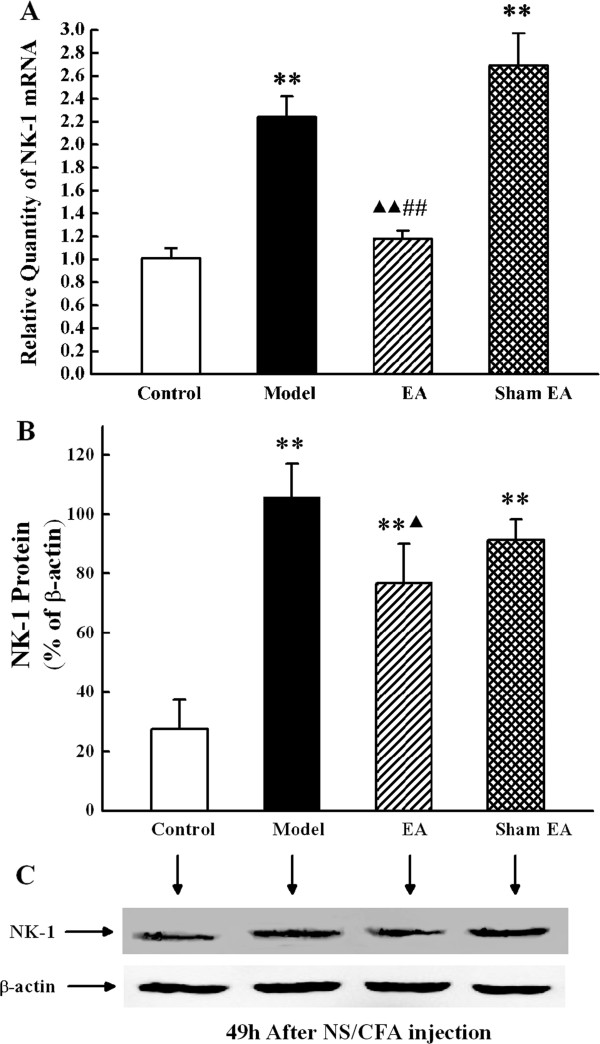
**Effect of EA on NK-1 expression in the ipsilateral spinal dorsal horn at 49 h after CFA injection. (A)**. The expression of NK-1 mRNA (relative to GAPDH) in L4-6 ipsilateral spinal dorsal horn measured by real-time PCR. **P < 0.01 versus control group. ^▲▲^ *P* < 0.01 versus model group. **##** *P* < 0.05 versus sham EA group. Results are mean ± SEM; n = 6. **(B)**. The expressions of NK-1 protein in the L4-6 ipsilateral spinal cord dorsal horn by western blotting analysis. **P < 0.01 versus control group. ^▲^ *P* < 0.05 versus model group. Results are mea n ± SEM; n = 6. **C**. NK-1 protein in normalized against β-actin.

Since NK-1 was over-expressed in SCDH at 49 h after CFA injection, the regulating effect of EA on NK-1 expression in SCDH was assessed by real-time PCR and western blot at this time point. Compared with the control group, expression level of NK-1 mRNA and protein in SCDH was significantly increased in the model group (*P* < 0.01). EA treatment significantly down-regulated the expression of NK-1 mRNA, compared with the control group (*P* < 0.01, Figure 5A). Similarly, EA also suppressed the CFA-induced increase of NK-1 protein expression in SCDH (*P* < 0.05, Figure 5B-C). Sham EA produced no inhibitory effect on NK-1 mRNA or protein expression (*P* > 0.05) (Figure 5).

### Peripheral inflammation has no effect on Elk1 expression

**Figure 6:**
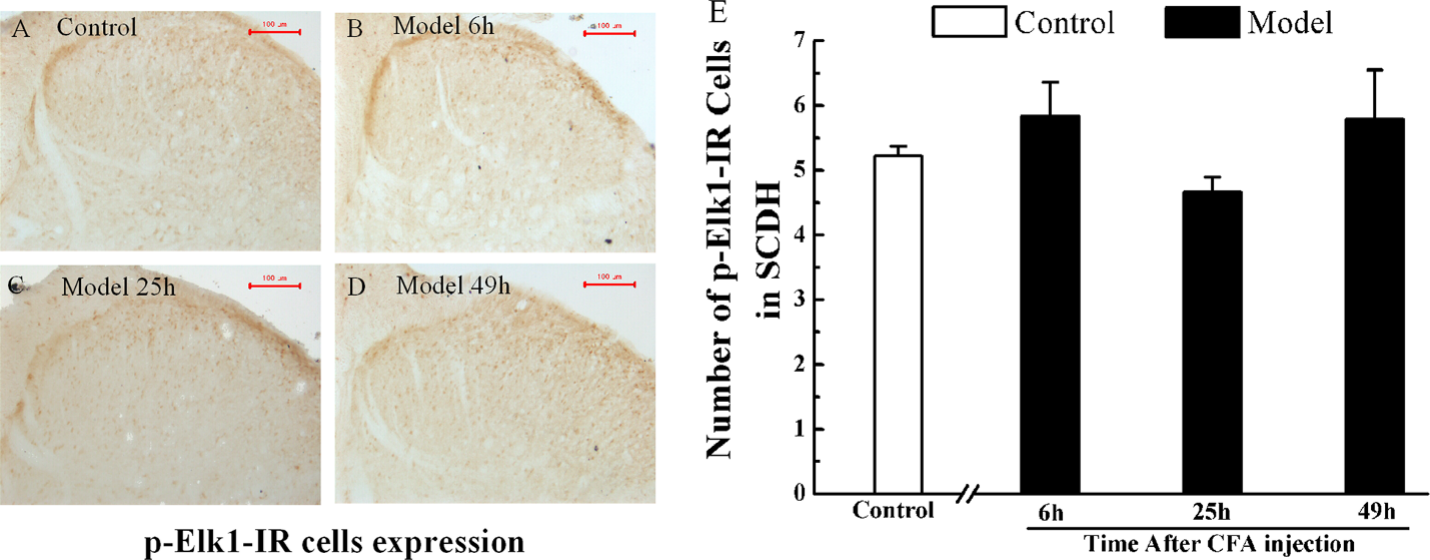
**The expression of p-Elk1-IR cells in spinal cord dorsal horn of rats in the control group and at 6, 25 and 49 h after subcutaneous injection of CFA into the plantar hind paw region. A-D**. Immunohistochemical analysis shows p-Elk1-IR cells in the L4-6 ipsilateral spinal cord dorsal horn of control rats **(A)**, model rats at 6 h **(B)**, 25 h **(C)** and 49 h **(D)**. **E**. Quantification of p-Elk1-IR cells in L4-6 ipsilateral spinal cord dorsal horn, respectively. Results are mean ± SEM; n = 6. *P < 0.05, **P < 0.01 versus control group at the corresponding time point. The scale bar is 100 μm.

Elk1 is a well-characterized downstream nuclear substrate of ERK1/2 and p-Elk1 tend to move into the nucleus to regulate target transcription factors. Since our study suggested a role for EA in inhibiting p-ERK1/2, COX-2 and NK-1 mRNA expression in CFA rats, the expression of p-Elk1 was measured in SCDH at each time point. However, the numbers of p-Elk1-IR cells in the model group were unaffected at each time point compared with the control group (P > 0.05) (Figure 6).

### Effect of EA on CREB combination

**Figure 7:**
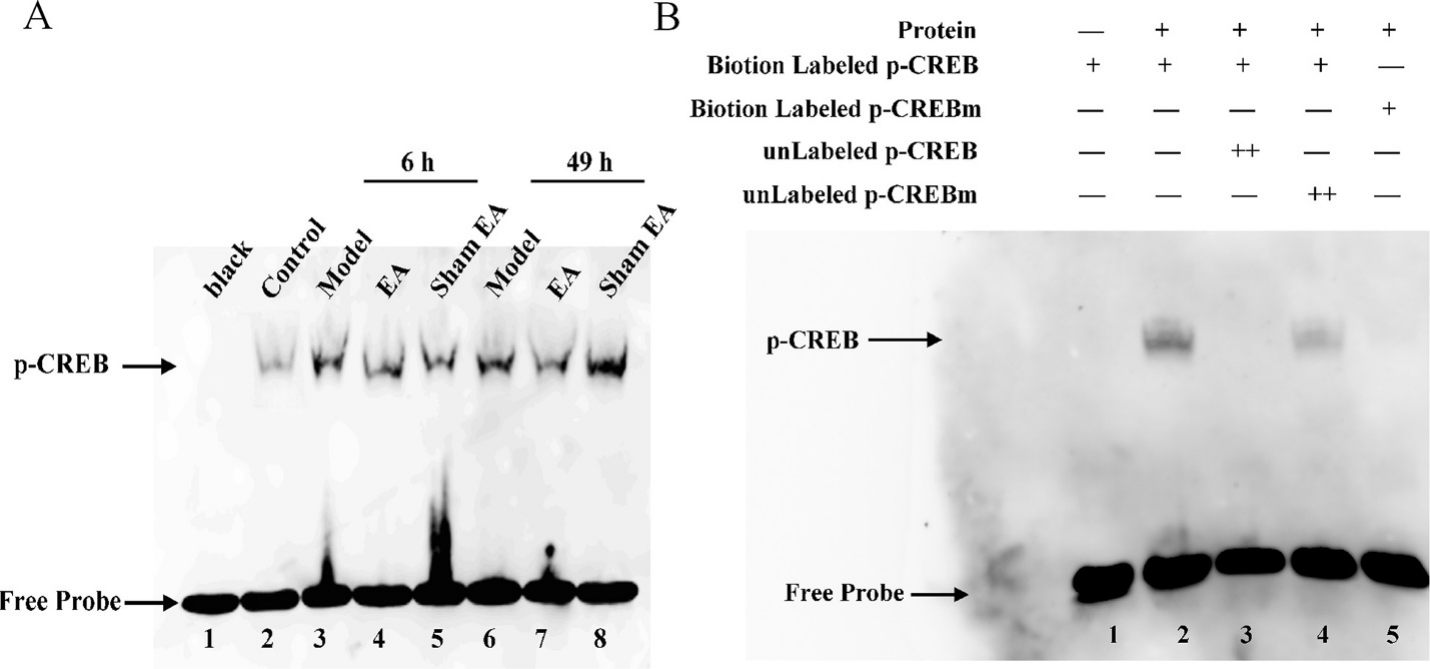
**Effect of EA on p-CREB DNA binding activity in L4-6 ipsilateral spinal cord dorsal horn. (A)**. EMSA experiment showing binding to CREB probe in nuclear extracts prepared from control and model, EA, and sham EA groups at 6 and 49 h after CFA injection. The binding of CREB in the control group was considered for comparison (lane 2). EA regulated CREB protein binding activity at 49 after CFA-treated (lane 7). **(B)**. EMSA experiment showing the binding of proteins present in the nuclear extracts prepared from the model group at 49 h after CFA injection. Specificity of the DNA-protein interaction was assessed by competition EMSA using a 100× molar excess of unlabeled doubled-stranded oligonucleotide CREB probe (lane 3), unlabeled mutant doubled-stranded oligonucleotide CREB probe (lane 4) and biotin-labeled mutant doubled-stranded oligonucleotide CREB probe (lane 5).

Since our study suggested a role for EA in inhibiting COX-2 and NK-1 expression in CFA rats, the nuclear extracts from SCDH of rats in the control, model, EA and sham EA groups at 6 and 49 h after CFA injection were analyzed. SCDH nuclear extracts from the control group did not contain appreciable levels of CREB-binding (Figure 7A, lane 2), whereas nuclear extracts from the model, EA and sham EA groups bound very efficiently to a CREB consensus probe (Figure 7A, lanes 3–8). In addition, the binding ability in the EA group at 49 h was weaker than that observed in the model and sham EA groups at the same time point (Figure 7A, lane 7). To check the specificity of the protein-DNA interactions, competitive EMSA was performed, using 100× molar excess of unlabeled doubled-stranded oligonucleotide CREB probe (unlabeled p-CREB) and unlabeled mutant doubled-stranded oligonucleotide CREB probe (unlabeled p-CREBm) added as a competitor for the respective treatments. No CREB binding to the target DNA was observed in the competitive assay of unlabeled p-CREB probe (Figure 7B, lane3) and weak binding was observed in the competitive assay using an unlabeled p-CREBm probe (Figure 7B, lane4). Similarly, biotin-labeled mutant doubled-stranded oligonucleotide CREB probe (biotin-labeled p-CREBm) was used to check the specificity of protein-DNA interactions. No binding was observed in the assay (Figure 7B, lane5).

### Analgesic effect of electroacupuncture plus U0126 on inflammatory pain

To elucidate whether ERK1/2 is involved in the analgesic effect of EA, U0126, a MEK inhibitor, was delivered intrathecally with EA treatment at 5, 24 and 48 h after CFA injection. As described above, the repeated-measures ANOVA with between-subjects factors also revealed differences over time points (*P* < 0.01) and between groups (*P* < 0.01). *Post-hoc* LSD tests indicated that, after CFA injection, both the administration of EA + DMSO and EA + U0126 increased rat PWTs compared with DMSO treatment alone (*P* < 0.01). Furthermore, PWTs in the EA + U0126 group showed a significant increase compared with the EA + DMSO group (*P* < 0.01).

**Figure 8:**
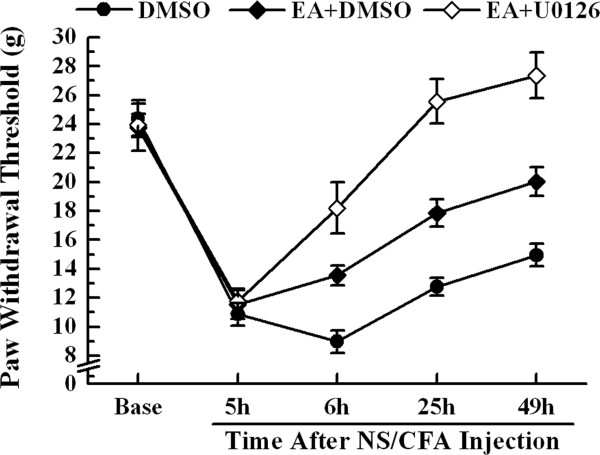
**Effect of MEK inhibitor (U0126) on PWTs to mechanical stimuli in CFA-injected rats with EA stimulation.** PWTs were measured at pre-injection, 5, 6, 25 and 49 h after CFA injection. Values represent mean ± SEM; n = 10 per group for each time point. ○○*P* < 0.01 versus DMSO group at the corresponding time point. △△*P* < 0.01 versus EA plus DMSO group at the corresponding time point.

One-way ANOVA for independent samples identified significant differences occurred between the DMSO, EA + DMSO and EA + U0126 groups from 6 to 49 h after CFA injection (*P* < 0.01). Administration of EA + DMSO increased the mechanical PWTs from 6 to 49 h after CFA injection compared to with the DMSO control group (p < 0.01). Moreover, the analgesic effect elicited by co-treatment of EA and U0126 was significantly stronger in mechanical allodynia at 6, 25 and 49 h after modeling when compared with EA or DMSO alone (*P* < 0.01) (Figure 8).

## Discussion

Although early studies of ERK1/2 focused on its relation with mitosis, proliferation and differentiation of cells [24], a growing body of evidence suggests that ERK1/2 activation contributes to pain hypersensitivity [25]. Especially, ERK1/2 activation in SCDH plays a key role in developing and maintaining mechanical allodynia induced by peripheral inflammation [26–29]. In the present study, p-ERK1/2 positive cells in the ipsilateral side of SCDH significantly increased during the developmental phase (6 to 49 h after CFA injection) of inflammatory pain-related hypersensitivity, consistent with published data from previous studies [12, 26]. However, results from western blot showed p-ERK1/2 protein only increased at the 6 h after CFA injection, not at 49 h. Because ERK is only activated in superficial dorsal horn (laminae I-II), western blot may be less sensitive and accuracy than immunohistochemistry in detecting ERK activation in such small subset of SCDH, and the result that the number of p-ERK1/2 positive cells at 49 h is much lesser than at 6 h after CFA injection may confirm this conjecture. Some reports revealed ERK1/2 activation could regulate the expression level of COX-2 and NK-1 [12, 13], two well-characterized mediators of pain, we investigated their expression further. COX-2 has been thought to contribute to inflammatory pain for many years [23, 30]. Our results showed that CFA induced mechanical allodynia and an increase in COX-2 protein and mRNA expression on the ipsilateral side of the SCDH at 6 h after injection. These mechanisms might allow COX-2 to perform as a central component of inflammatory pain hypersensitivity in neurons of the central nervous system by increasing neuronal excitation and reducing neuronal inhibition [31]. Several evidence also suggested that NK-1 in the SCDH played an important role in inflammatory pain hypersensitivity [32]. The amount and internalization of NK-1 receptors on SCDH neuron densities increased after peripheral inflammation [33], and induced mechanical allodynia in several inflammatory pain models [34, 35]. In agreement with previous studies [12, 36], peripheral inflammation up-regulated NK-1 mRNA and protein in SCDH at 49 h after CFA injection. Taken together, we hypothesized that ERK1/2 activation after peripheral inflammation resulted in pain hypersensitivity by inducing different protein expression at different time points in CFA rats. Activated ERK1/2 induced COX-2 expression at 6 h after CFA injection and NK-1 expression at 49 h after CFA injection. However, we did not observe either COX-2 or NK-1 over-expressions at 25 h after CFA treatment. The reason for this is not clear and requires further study.

EA treatment has been shown to have analgesic effects in various pain models [37–41]. In the present study, EA significantly increased rat PWTs after a single treatment (6 h after CFA injection) and maintained the analgesic effect by repeated administration during the whole study period. Moreover, in addition to the analgesic effect, EA treatment significantly inhibited ERK1/2 activation in SCDH. These results, similar as we reported before [19], delineated the crucial role of ERK1/2 in the analgesic effect of EA. Since TENS, mostly thought has the same mechanism with EA, produced analgesic effect by regulated ERK1/2 signaling pathway, we further investigated EA’s effect on the downstream substance of ERK1/2 [18]. Previous studies demonstrated that EA treatment produced anti-inflammatory pain effects through the regulation of COX-2 and NK-1 expression in both peripheral and central nociceptive sites, which were induced by peripheral inflammation [42–44]. Along with the change of ERK1/2 activation levels, EA significantly suppressed COX-2 protein expression, but not COX-2 mRNA expression at 6 h after CFA injection. It is generally thought that effects produced by transcriptional regulation should take a relatively long of time [12]. In this experiment, the therapeutic effect produced by inhibiting ERK1/2 activation might be too quick (<1 h) to be mediated by the inhibition of transcription and therefore it likely represented a post-translational change of ERK1/2, similar as previous study [11]. So, the analgesic effect of EA might be partly produced by regulating post-translational changes. On the other hand, the effect of EA on NK-1 expression was detected. Different to the regulatory effects on COX-2, EA prevented increased expression of NK-1 mRNA and protein in SCDH at 49 h after CFA injection. Since EA regulated p-ERK1/2 and NK-1 in a time-dependent way, we hypothesized that the EA analgesic effect was also produced by regulating transcription changes. Thus, EA treatment may regulate ERK1/2-COX-2 protein at 6 h after CFA injection and ERK1/2-NK-1 mRNA/protein at 49 h after CFA injection.

Activated ERK1/2, an intracellular signaling molecule, is translocated to the nucleus to phosphorylate downstream nuclear substrates such as Elk1 [16, 45]. A previous study demonstrated that Elk1 formed an important link in the ERK pathway to transduce signals from the cell surface to the nucleus for the activation of genetic machinery necessary to maintain synaptic plasticity [46]. Synaptic plasticity is regarded as the foundation of central sensitization and pain. However, we did not observe a difference in p-Elk1 expression between the control and model groups at any time point studied. This indicated that ERK1/2 might not regulate gene transcription via activating Elk1 in SCDH in CFA rats.

We also tested the DNA binding activity of CREB, another downstream transcriptional target of activated ERK1/2. Noxious stimulation and peripheral inflammation evoked CREB activation in the dorsal horn [47]. Similarly, we found the DNA binding activity of CREB was up-regulated by peripheral inflammation at 6 and 49 h after CFA injection. EA treatment significantly down-regulated the DNA binding activity of CREB at 49 h after CFA injection, but not at 6 h. Since several studies demonstrated phospho-CREB (p-CREB) could transactivate COX-2 and NK-1 promoters [48, 49], ERK1/2 activation might contribute to inflammatory pain by the ERK1/2-CREB-NK-1 pathways in SCDH. This result is compatible with the above findings that EA alleviated inflammatory pain by post-translational regulation at 6 h and by transcriptional regulation at 49 h. Therefore, inhibition of ERK1/2-COX-2 pathway activation by post-translational regulation and ERK1/2-CREB-NK-1 pathway activation by transcriptional regulation may be two potential methods to alleviate persistent inflammatory pain by EA.

Since we previously demonstrated that EA regulate p38 MAPK pathway in the SCDH of inflammatory rats [21], and p-ERK1/2 was only partially inhibited by EA from the present finding, ERK pathway cannot be the only signal transduction pathway under EA analgesia. To confirm this, the effect of a MEK inhibitor administrated by intra-thecal injection, on CFA-induced hyperalgesic behavior was performed. EA plus U0126 showed a synergistic effect on antinociception against inflammatory pain. So EA alleviated inflammatory pain partly due to the regulation of ERK pathway in SCDH. There was a big difference of PWT between Figures 1 and 8. The main reason may be attributed to the conditions of rats, which were put into use in two parts of experiment, used in different batches, feed in different climates and tested in different rooms (though the temperatures being strictly controlled). The operation implanted the polyethylene-10 catheter into the intrathecal space of the spinal cord may be the second reason. However, the injection of CFA also induced a significant inflammatory pain, so we thought the results of Figure 8 are credible.

## Conclusions

In summary, the present study shows EA treatment might be a useful therapy for the treatment of inflammatory pain allodynia. This analgesic effect may be associated with inhibition of ERK pathway activation in SCDH. Since activated ERK1/2 up-regulated COX-2 and NK-1 at different time points, different mechanisms exist in the mechanism of EA-induced analgesia, including post-translational regulation-induced inhibition of ERK1/2-COX-2 pathway activation, or transcriptional regulation-induced inhibition of ERK1/2-CREB-NK-1 pathway activation.

## References

[1] Carter GT, Duong V, Ho S, Ngo KC, Greer CL, Weeks DL (2014). Side effects of commonly prescribed analgesic medications. Phys Med Rehabil Clin N Am.

[2] Han JS (2011). Acupuncture analgesia: areas of consensus and controversy. Pain.

[3] Zhao ZQ (2008). Neural mechanism underlying acupuncture analgesia. Prog Neurobiol.

[4] Zhang R, Lao L, Ren K, Berman BM (2014). Mechanisms of acupuncture-electroacupuncture on persistent pain. Anesthesiology.

[5] Liang Y, Fang JQ, Du JY, Fang JF (2012). Effect of electroacupuncture on activation of p38MAPK in spinal dorsal horn in rats with complete Freund’s adjuvant-induced inflammatory pain. Evid Based Complement Alternat Med.

[6] Suarez-Almazor ME, Looney C, Liu Y, Cox V, Pietz K, Marcus DM, Street RL (2010). A randomized controlled trial of acupuncture for osteoarthritis of the knee: effects of patient-provider communication. Arthritis Care Res (Hoboken).

[7] Ji RR, Samad TA, Jin SX, Schmoll R, Woolf CJ (2002). p38 MAPK activation by NGF in primary sensory neurons after inflammation increases TRPV1 levels and maintains heat hyperalgesia. Neuron.

[8] Doya H, Ohtori S, Fujitani M, Saito T, Hata K, Ino H, Takahashi K, Moriya H, Yamashita T (2005). c-Jun N-terminal kinase activation in dorsal root ganglion contributes to pain hypersensitivity. Biochem Biophys Res Commun.

[9] Ji RR, Woolf CJ (2001). Neuronal plasticity and signal transduction in nociceptive neurons: implications for the initiation and maintenance of pathological pain. Neurobiol Dis.

[10] Lee KM, Kang BS, Lee HL, Son SJ, Hwang SH, Kim DS, Park JS, Cho HJ (2004). Spinal NF-kB activation induces COX-2 upregulation and contributes to inflammatory pain hypersensitivity. Eur J Neurosci.

[11] Ji RR, Baba H, Brenner GJ, Woolf CJ (1999). Nociceptive-specific activation of ERK in spinal neurons contributes to pain hypersensitivity. Nat Neurosci.

[12] Ji RR, Befort K, Brenner GJ, Woolf CJ (2002). ERK MAP kinase activation in superficial spinal cord neurons induces prodynorphin and NK-1 upregulation and contributes to persistent inflammatory pain hypersensitivity. J Neurosci.

[13] Lonze BE, Ginty DD (2002). Function and regulation of CREB family transcription factors in the nervous system. Neuron.

[14] Malmberg AB, Yaksh TL (1992). Hyperalgesia mediated by spinal glutamate or substance P receptor blocked by spinal cyclooxygenase inhibition. Science.

[15] Traub RJ (1996). The spinal contribution of substance P to the generation and maintenance of inflammatory hyperalgesia in the rat. Pain.

[16] Uht R, Amos S, Martin P, Riggan A, Hussaini I (2006). The protein kinase C-η isoform induces proliferation in glioblastoma cell lines through an ERK/Elk-1 pathway. Oncogene.

[17] Kawasaki Y, Kohno T, Zhuang ZY, Brenner GJ, Wang H, Van Der Meer C, Befort K, Woolf CJ, Ji RR (2004). Ionotropic and metabotropic receptors, protein kinase A, protein kinase C, and Src contribute to C-fiber-induced ERK activation and cAMP response element-binding protein phosphorylation in dorsal horn neurons, leading to central sensitization. J Neurosci.

[18] Fang JF, Liang Y, Du JY, Fang JQ (2013). Transcutaneous electrical nerve stimulation attenuates CFA-induced hyperalgesia and inhibits spinal ERK1/2-COX-2 pathway activation in rats. BMC Complement Altern Med.

[19] Jian-qiao F, Jun-fan F, Yi L, Jun-ying D, Yu-jie Q, Jing L (2012). Immediately analgesic effect of electroacupuncture and its mechanism via spinal p-ERK1/2. Chin Acupunct Moxibustion.

[20] Cheng CY, Lin JG, Su SY, Tang NY, Te Kao S, Hsieh CL (2014). Electroacupuncture-like stimulation at baihui and dazhui acupoints exerts neuroprotective effects through activation of the brain-derived neurotrophic factor-mediated MEK1/2/ERK1/2/p90RSK/bad signaling pathway in mild transient focal cerebral ischemia in rats. BMC Complement Altern Med.

[21] Fang JQ, Du JY, Liang Y, Fang JF (2013). Intervention of electroacupuncture on spinal p38 MAPK/ATF-2/VR-1 pathway in treating inflammatory pain induced by CFA in rats. Mol Pain.

[22] Yamamoto T, Sakashita Y (1999). The role of the spinal opioid receptor like1 receptor, the NK-1 receptor, and cyclooxygenase-2 in maintaining postoperative pain in the rat. Anesth Analg.

[23] Samad TA, Moore KA, Sapirstein A, Billet S, Allchorne A, Poole S, Bonventre JV, Woolf CJ (2001). Interleukin-1beta-mediated induction of Cox-2 in the CNS contributes to inflammatory pain hypersensitivity. Nature.

[24] Widmann C, Gibson S, Jarpe MB, Johnson GL (1999). Mitogen-activated protein kinase: conservation of a three-kinase module from yeast to human. Physiol Rev.

[25] Ji RR, Gereau RW, Malcangio M, Strichartz GR (2009). MAP kinase and pain. Brain Res Rev.

[26] Adwanikar H, Karim F, Gereau RW (2004). Inflammation persistently enhances nocifensive behaviors mediated by spinal group I mGluRs through sustained ERK activation. Pain.

[27] Cruz CD, Avelino A, McMahon SB, Cruz F (2005). Increased spinal cord phosphorylation of extracellular signal-regulated kinases mediates micturition overactivity in rats with chronic bladder inflammation. Eur J Neurosci.

[28] Karim F, Wang CC, Gereau RW (2001). Metabotropic glutamate receptor subtypes 1 and 5 are activators of extracellular signal-regulated kinase signaling required for inflammatory pain in mice. J Neurosci.

[29] Pang XY, Liu T, Jiang F, Ji YH (2008). Activation of spinal ERK signaling pathway contributes to pain-related responses induced by scorpion Buthus martensi Karch venom. Toxicon.

[30] Yaksh TL, Dirig DM, Conway CM, Svensson C, Luo ZD, Isakson PC (2001). The acute antihyperalgesic action of nonsteroidal, anti-inflammatory drugs and release of spinal prostaglandin E2 is mediated by the inhibition of constitutive spinal cyclooxygenase-2 (COX-2) but not COX-1. J Neurosci.

[31] Vardeh D, Wang D, Costigan M, Lazarus M, Saper CB, Woolf CJ, Fitzgerald GA, Samad TA (2009). COX2 in CNS neural cells mediates mechanical inflammatory pain hypersensitivity in mice. J Clin Invest.

[32] Honoré P, Menning PM, Rogers SD, Nichols ML, Basbaum AI, Besson JM, Mantyh PW (1999). Spinal substance P receptor expression and internalization in acute, short-term, and long-term inflammatory pain states. J Neurosci.

[33] Abbadie C, Trafton J, Liu H, Mantyh PW, Basbaum AI (1997). Inflammation increases the distribution of dorsal horn neurons that internalize the neurokinin-1 receptor in response to noxious and non-noxious stimulation. J Neurosci.

[34] Trafton JA, Basbaum AI (2000). The contribution of spinal cord neurokinin-1 receptor signaling to pain. J Pain.

[35] Woolf CJ, Mannion RJ, Neumann S (1998). Null mutations lacking substance: elucidating pain mechanisms by genetic pharmacology. Neuron.

[36] McCarson KE, Krause JE (1994). NK-1 and NK-3 type tachykinin receptor mRNA expression in the rat spinal cord dorsal horn is increased during adjuvant or formalin-induced nociception. J Neurosci.

[37] Kim SK, Park JH, Bae SJ, Kim JH, Hwang BG, Min BI, Park DS, Na HS (2005). Effects of electroacupuncture on cold allodynia in a rat model of neuropathic pain: mediation by spinal adrenergic and serotonergic receptors. Exp Neurol.

[38] Xing GG, Liu FY, Qu XX, Han JS, Wan Y (2007). Long-term synaptic plasticity in the spinal dorsal horn and its modulation by electroacupuncture in rats with neuropathic pain. Exp Neurol.

[39] Baek YH, Choi DY, Yang HI, Park DS (2005). Analgesic effect of electroacupuncture on inflammatory pain in the rat model of collagen-induced arthritis: mediation by cholinergic and serotonergic receptors. Brain Res.

[40] Huang C, Hu ZP, Long H, Shi YS, Han JS, Wan Y (2004). Attenuation of mechanical but not thermal hyperalgesia by electroacupuncture with the involvement of opioids in rat model of chronic inflammatory pain. Brain Res Bull.

[41] Sekido R, Ishimaru K, Sakita M (2004). Corticotropin-releasing factor and interleukin-1beta are involved in the electroacupuncture-induced analgesic effect on inflammatory pain elicited by carrageenan. Am J Chin Med.

[42] Zhang RX, Liu B, Qiao JT, Wang L, Ren K, Berman BM, Lao L (2005). Electroacupuncture suppresses spinal expression of neurokinin-1 receptors induced by persistent inflammation in rats. Neurosci Lett.

[43] Fang J, Aoki E, Yu Y, Sohma T, Kasahara T, Hisamitsu T (1999). Inhibitory effect of electroacupuncture on murine collagen arthritis and its possible mechanisms. In Vivo.

[44] Lee JH, Jang KJ, Lee YT, Choi YH, Choi BT (2006). Electroacupuncture inhibits inflammatory edema and hyperalgesia through regulation of cyclooxygenase synthesis in both peripheral and central nociceptive sites. Am J Chin Med.

[45] Hsieh HL, Wu CY, Yang CM (2008). Bradykinin induces matrix metalloproteinase‐9 expression and cell migration through a PKC‐δ‐dependent ERK/Elk‐1 pathway in astrocytes. Glia.

[46] Davis S, Vanhoutte P, Pagès C, Caboche J, Laroche S (2000). The MAPK/ERK cascade targets both Elk-1 and cAMP response element-binding protein to control long-term potentiation-dependent gene expression in the dentate gyrus in vivo. J Neurosci.

[47] Messersmith DJ, Kim DJ, Iadarola MJ (1998). Transcription factor regulation of prodynorphin gene expression following rat hindpaw inflammation. Mol Brain Res.

[48] Eliopoulos AG, Dumitru CD, Wang CC, Cho J, Tsichlis PN (2002). Induction of COX-2 by LPS in macrophages is regulated by Tpl2-dependent CREB activation signals. EMBO J.

[49] Duric V, McCarson KE (2007). Neurokinin-1 (NK-1) receptor and brain-derived neurotrophic factor (BDNF) gene expression is differentially modulated in the rat spinal dorsal horn and hippocampus during inflammatory pain. Mol Pain.

[CR50] The pre-publication history for this paper can be accessed here:http://www.biomedcentral.com/1472-6882/14/285/prepub

